# P187: Harnessing the power of the mind to reduce healthcare associated infections - a cost effective approach in low resource settings

**DOI:** 10.1186/2047-2994-2-S1-P187

**Published:** 2013-06-20

**Authors:** N Jaggi, P Nirwan, E Naryana, KP Kaur

**Affiliations:** 1 Infection control , Artemis Health Insitute, Gurgaon, India

## Introduction

Infection prevention is a mind set and the reasons for non compliance are related to psychological barriers, preconceived notions, cultural influences and ineffective time management rather than lack of available resources or knowledge. Our mind is the most powerful tool. Can we harness the power of the mind in oneself and others to understand the psychology of non compliance as also ways to improve implementation of infection control practices and subsequently reduce healthcare associted infections ( HAI's ).

## Objectives

To expand the skill set of infection preventionists as a cost effective approach.

## Methods

This study was initiated in July 2012 and lasted for 6 months till December 2012. Twelve infection control team leaders were identified from each department after conducting a basic technical and psychological assessment .An advanced psychological assessment was then performed on them A trained psychologist was employed to impart relevant soft skill training to them focusing on harnessing the power of the mind to effectively manage their time as also collaborate with other teams to achieve the desired result .The training involved didactic lectures as well as simulation to teach real life skills .A post assessment was conducted after training and results statistically analyzed. The compliance to infection control guidelines and healthcare associated infections ( HAI’s ) identified in each of their units were then correlated with the increase in their behavioral competencies.

## Results

10 of the 12 (83.3%) showed significant improvement in all aspects of competency in infection prevention. The chief criterion included were powers of negotiation, ability to get along with peers, juniors and seniors, leadership skills, communication skills and emotional intelligence. There was a significant increase (p<0..05) in all separate parameters considered for assessment. A positive correlation was observed between the compliance to infection control guidelines and HAI’s with the increase in competencies.

## Conclusion

Focusing on power of the mind and improving psychological competencies in infection preventionists can lead to a cost effective and rational approach to increasing compliance to guidelines and reduce the HAI’s.

## Disclosure of interest

None declared

